# Virtual Screening and Structure-Based Discovery of Indole Acylguanidines as Potent β-secretase (BACE1) Inhibitors

**DOI:** 10.3390/molecules18055706

**Published:** 2013-05-16

**Authors:** Yiquan Zou, Li Li, Wuyan Chen, Tiantian Chen, Lanping Ma, Xin Wang, Bing Xiong, Yechun Xu, Jingkang Shen

**Affiliations:** 1 State Key Laboratory of Drug Research, Shanghai Institute of Materia Medica, Chinese Academy of Sciences, 555 Zuchongzhi Road, Zhangjiang Hi-Tech Park, Shanghai 201203, China; E-Mails: zyq2047@163.com (Y.Z.); lpma@simm.ac.cn (L.M.); wangxin@simm.ac.cn (X.W.); 2 CAS Key Laboratory of Receptor Research, Shanghai Institute of Materia Medica, Chinese Academy of Sciences, 555 Zuchongzhi Road, Zhangjiang Hi-Tech Park, Shanghai 201203, China; E-Mails: li-li@simm.ac.cn (L.L.); wychen@simm.ac.cn (W.C.); ttchen@simm.ac.cn (T.C.)

**Keywords:** virtual screening, docking, structure-based lead design, crystal structure, indole acylguanidine

## Abstract

Proteolytic cleavage of amyloid precursor protein by β-secretase (BACE1) is a key step in generating the *N*-terminal of β-amyloid (Aβ), which further forms into amyloid plaques that are considered as the hallmark of Alzheimer’s disease. Inhibitors of BACE1 can reduce the levels of Aβ and thus have a therapeutic potential for treating the disease. We report here the identification of a series of small molecules bearing an indole acylguanidine core structure as potent BACE1 inhibitors. The initial weak fragment was discovered by virtual screening, and followed with a hit-to-lead optimization. With the aid of co-crystal structures of two discovered inhibitors (compounds **19** and **25**) with BACE1, we explored the SAR around the indole and aryl groups, and obtained several BACE1 inhibitors about 1,000-fold more potent than the initial fragment hit. Accompanying the lead optimization, a previously under-explored sub-site opposite the flap loop was redefined as a potential binding site for later BACE1 inhibitor design.

## 1. Introduction

Alzheimer’s disease (AD), characterized by the slow but inexorable loss of memory and cognition, is affecting nearly 30 million people worldwide [1]. The incidence of AD cases is expected to escalate dramatically in the coming decades in conjunction with the increase of life expectancy. In the past two decades, genetics, biochemistry, cell biology, animal models have been combined to provide a large body of evidences that, pathologically, AD is represented by the progressive formation of insoluble plaques formed by amyloid β-protein and fibrillary tangles related to hyperphosphorylated tau protein [2,3]. The amyloid β-peptide (Aβ), usually containing 40–42 amino acids, is derived from the β-amyloid precursor protein (APP) by subsequent proteolyses processed by β-site cleaving enzyme (BACE1) and γ-secretase [4,5]. This amyloid hypothesis is embraced by many researchers, and considerable efforts have been devoted to develop new medicines to cure, if not at least halt, the progress of the disease in AD patients [6,7]. 

Based on the amyloid hypothesis, BACE1 is recognized as an important drug target since it is the rate-limited enzyme during the production of Aβ and initializes the cleavage of APP. Besides, BACE1 knockout mice are viable and the transgenic mouse models show a clear sign of reduction in AD-like pathology by shutting down the activity of BACE1 [8,9,10,11]. 

Structurally, BACE1 belongs to the aspartyl protease family, and contains a bilobal structure forming by an N- and a C-terminal domains [12]. Both N- and C-domains are formed by highly twisted β-sheet structures and each domain contributes an aspartic acid to the catalytic module of the enzyme. It is thus proposed that ligands containing a positive charged moiety might be favorable to counteract the negative charged active site.

**Figure 1:**
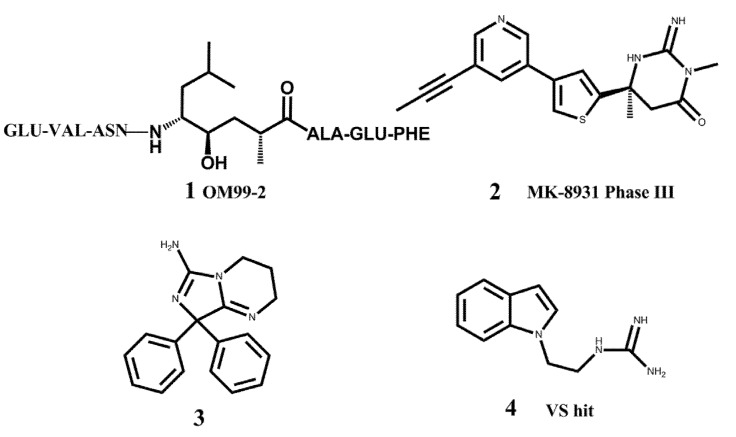
Representative BACE1 inhibitors.

In the past, major efforts in designing BACE1 inhibitors were focused on the transition state analogs such as hydroxyethylamines [13,14,15], hydroxyethylene [16,17], and statine-based peptidomimetic inhibitors [18,19]. Although a large number of potent peptidomimetic inhibitors have been discovered, their relatively large sizes and excessive number of hydrogen-bond donors and acceptors make it difficult for them to penetrate the blood brain barrier [20,21]. Therefore many researchers in both academia and industry are trying to identify drug-like small molecules as BACE1 inhibitors, which hold great hopes to have good pharmacokinetic (PK) profiles and are suitable for drug development. Besides the efforts of hit identification with high-throughput screening techniques, fragment-based drug discovery (FBDD) is considered a promising approach for hit identification. Godemann *et al.* employed a functional assay-based method to screen a diverse fragment library of 20,000 compounds, and obtained 26 novel hits for further drug development [22]. Congreve *et al.* at Astex applied their unique FBDD platform to BACE1, obtaining several fragment hits containing an aminopyridine motif [23]. They further carried out the lead optimization which led to a potent compound with an IC_50_ value of about 0.69 μM. Other pharmaceutical companies also launched similar FBDD projects to develop BACE1 inhibitors, such as the 6-substituted isocytosines discovered at AstraZeneca [24], and cyclic acylguanidines at Schering-Plough [25]. Currently, the most advanced compound targeting BACE1 in clinical trials is MK-8931 from Merck (**2** in Figure 1) [26]. 

In the present work, we first applied a docking-based virtual screening of a fragment library and found one small compound (**4** in Figure 1) with a weak inhibition activity towards BACE1. After a rational design based on the ligand-BACE1 co-crystal structures, we were able to synthesize a series of compounds bearing a core indole acylguanidine motif, several of which show low nanomolar inhibitions in enzymatic assays. As a consequence of this study, a druggable subpocket which is under-explored in the previous structure-activity relationship (SAR) studies on small molecular BACE1 inhibitors, was redefined. Together, we hope the results presented here can stimulate other researchers to develop new BACE1 inhibitors for AD treatment.

## 2. Results and Discussion

Finding novel compounds as starting points for lead optimization is a major challenge in drug discovery. In the present work, we were interested in identifying low molecular-weight fragments which usually have weak binding affinities in a range of 0.1–10 mM, but have high ligand efficiency. As demonstrated in many drug discovery projects, the fragment-based drug design approach has its strengths in obtaining drug candidates with a good PK profile, because the starting fragment has large room for further optimization of both the potency and the pharmacokinetic properties.

### 2.1. Virtual Screening

A virtual screening campaign on the ZINC fragment library (http://zinc.docking.org) was performed to identify suitable small fragments as the starting point. Firstly, from analysis of structures of ligand- bound BACE1 in the PDB database, it was found that the enzyme is flexible and can change its conformation according to the bound ligand, especially at the flap loop part. Based on the structural clustering results, we selected two structures (pdb entry ID: 1FKN and 3IGB) as the representatives to prepare the binding site models for the docking-based virtual screening [27,28]. In the structure 1FKN, BACE1 is bound with a landmark peptidemimic inhibitor OM99-2 (1); while 3IGB contains a small molecule bearing an aminoimidazole core in the binding site of BACE1 (3). Due to the binding of very different ligands, distinct conformations of the binding site, especially at the flap range, occurred in the two structures. The Schrödinger software package 7.5 was used to prepare the models for docking according to the standard protocol and default parameters of Glide. One hundred hits resulting from the docking were subjected to visualization of their binding orientations in the active site of BACE1. Five compounds were then purchased from a commercial vendor and tested with an enzymatic inhibition assay. One of the compounds, 1-(2-(1*H*-indol-1-yl)ethyl)guanidine (ZINC code: ZINC0 3650699, 4), showed weak inhibition activity towards BACE1, about 42% inhibition ratio at the ligand concentration of 100 μM in the fluorescence resonance energy transfer (FRET) assay system. 

### 2.2. Hit-to-Lead Optimization

**Figure 2:**
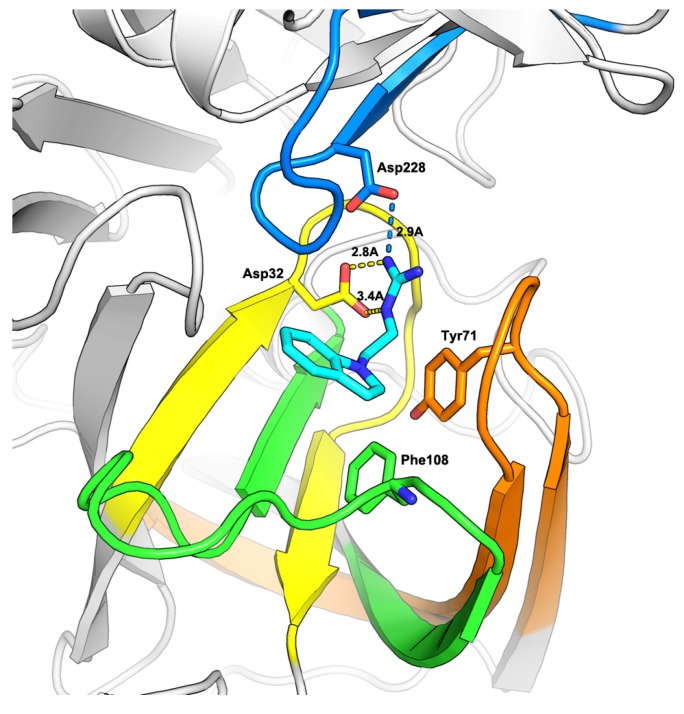
Docking study on compound **4** bound to BACE1. The coordinates of BACE1 was taken from the crystal structure of 1FKN. The protein is shown in cartoon, while the important residues and ligand **4** are shown in stick model.

As predicted by the Glide program, compound **4** occupied the S1 pocket and the guanidine moiety formed key binding interactions with the two catalytic aspartic acids, Asp32 and Asp228 (Figure 2). As exemplified in some known BACE1 inhibitors in which the guanidine group is usually acylated [29,30], we further designed a compound by introducing a carbonyl group into the α-position of the guanidine moiety. 

**Scheme 1:**
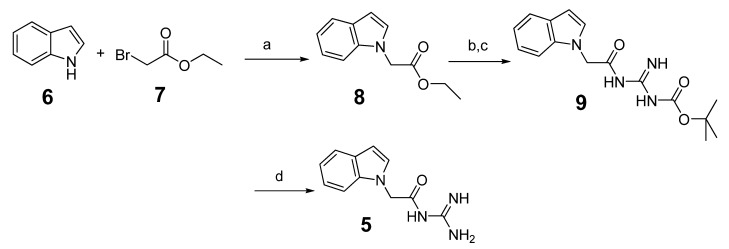
The synthesis of compound **5**.

The synthesis route is depicted in Scheme 1. Indole (**6**) was reacted with ethyl bromoacetate (7) in refluxing acetone in the presence of potassium carbonate to obtain ethyl 2-(1*H*-indol-1-yl) acetate (**8**). Then hydrolysis and condensation produced the intermediate **9** [31,32]. Intermediate **9** was de-protected by 4M HCl to afford compound **5**.

After the BACE1 enzymatic inhibition assay, it was found that the potency of compound **5** was improved to 77% inhibition ratio at the concentration of 100 µM.

*Reagents and conditions*: (**a**) K_2_CO_3_, acetone, reflux 5 h; (**b**) NaOH, THF/EtOH/H_2_O=2/2/1, reflux, 1 h; (c) HATU, NMM, r.t.; (d) 4M HCl in dioxane.

**Scheme 2:**
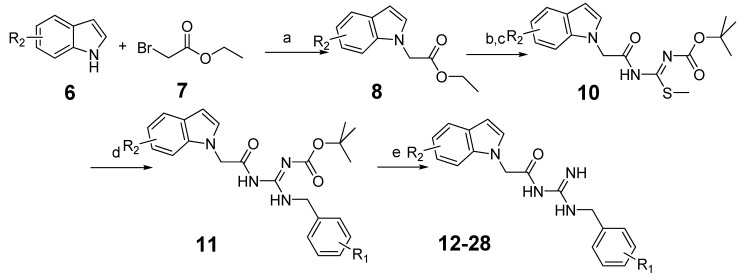
The synthesis of benzyl analogs.

To further improve the activity of this series of indole acylguanidines toward BACE1, we scrutinized the predicted conformation of compound **5** in the binding site of BACE1. As seen in Figure 2, there is a large hydrophobic sub-site at the top of the guanidine moiety. We speculated that a benzyl group extending from the terminus of the guanidine moiety could fill this sub-pocket and thereby potentially increase the binding affinity. Initial SAR investigation along this direction was conducted. The analogs were synthesized based on indole and ethyl bromoacetate using a method shown in Scheme 2.

*Reagents and conditions*: (**a**) K_2_CO_3_, acetone, reflux, 5 h; (**b**) NaOH, THF/EtOH/H_2_O=2/2/1, reflux, 1 h; (c) HATU, NMM, r.t.; (**d**) amine, Et_3_N, DCM, r.t. overnight; (**e**) 4M HCl in dioxane.

Briefly, indole (**6**) was reacted with ethyl bromoacetate (**7**) as before to produce **8** [30]. Then through hydrolysis and condensation isothiourea intermediates **10** were obtained [31,32,33]. The methylthio- group could be easily displaced by benzylamine [34,35,36], and further followed by acid mediated deprotection the target substituted benzylguanidine compounds were generated. All the compounds were tested by the BACE1 enzymatic inhibition assay at the concentration of 100 µM, and IC_50_ values were determined for compounds showing > 90% inhibition to the enzyme (Table 1).

**Table 1:** BACE1 inhibitory activities of compounds **12**–**21**. 
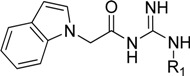

Comp.	R_1_	Inhibition ratio (100 μM)	IC_50_ (μM)
5	H	77.00	ND ^a^
12	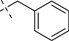	40.69	ND
13	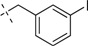	85.24	ND
14	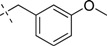	58.75	ND
15	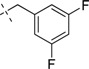	52.38	ND
16	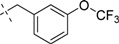	66.51	ND
17	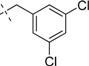	100	8.750 ± 1.075
18	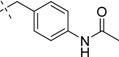	35.3	ND
19	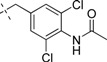	100	1.010 ± 0.091
20	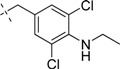	46.8	ND
21	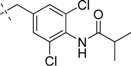	92.2	1.720 ± 0.210

^a^ ND means not determined.

**Figure 3:**
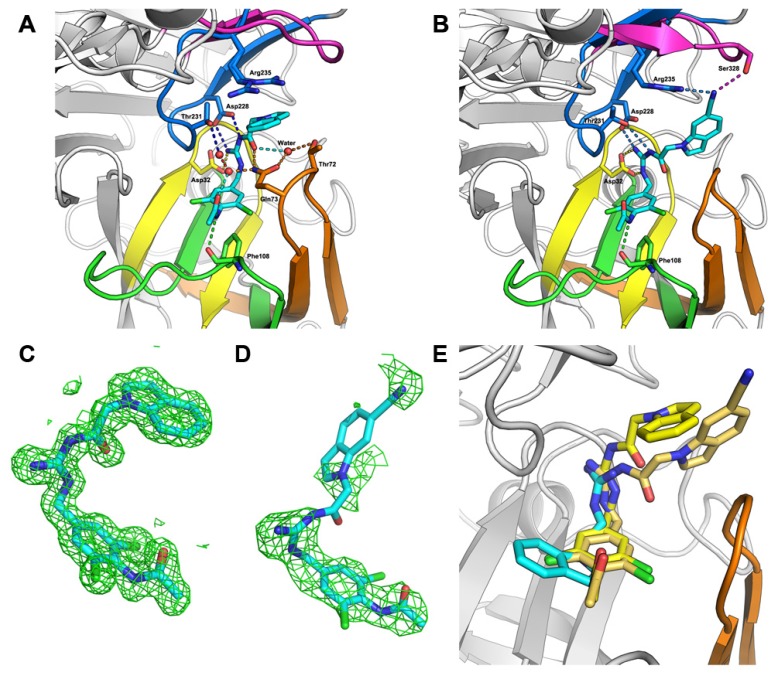
The structures of BACE1 in complex with compounds. (**A**–**B**) Cartoon representation of the crystal structure of BACE1 in complex with compounds **19** (**A**) and **25** (**B**). The pdb codes for generating figures A and B are 4IVT and 4IVS, respectively. The key residues and ligands **19** and **25** are highlighted with sticks. (**C**–**D**) (*F_o_*− *F_c_*) difference electron-density maps contoured at 2.0 σ for compounds **19** (**C**) and **25** (**D**). (**E**) The superimposition of the structures of BACE1 in complex with the compounds **19**, **25** and the docked fragment. Three small molecules were shown with sticks.

As shown in Table 1, the introduction of the simple benzyl group into **5** did not enhance the inhibitory activity. However, when a 3,5-dichlorobenyl group was introduced (compound **17**), the potency was improved and the corresponding IC_50_ value was 8.75 µM. To our surprise, compound **19** (IC_50_ = 1.01 µM), having an acetamide group between two chlorine atoms, showed a more substantial increase in potency than compound **4**. When we replaced two chlorine atoms with two hydrogen atoms or reduced the amide to an amine (compounds **18**, **20**), the resulting compounds displayed much weaker activities against BACE1. To characterize the binding mode of indole acylguanidines to BACE1, we used X-ray crystallography to determinate the *bona fide* conformation of ligands bound to the enzyme (Figure 3). Fortunately, the crystal structure of compound **19** in complex with the catalytic domain of human BACE1 could be determined successfully at the resolution of 1.6 Å (Figure 3A,C).

**Figure 4:**
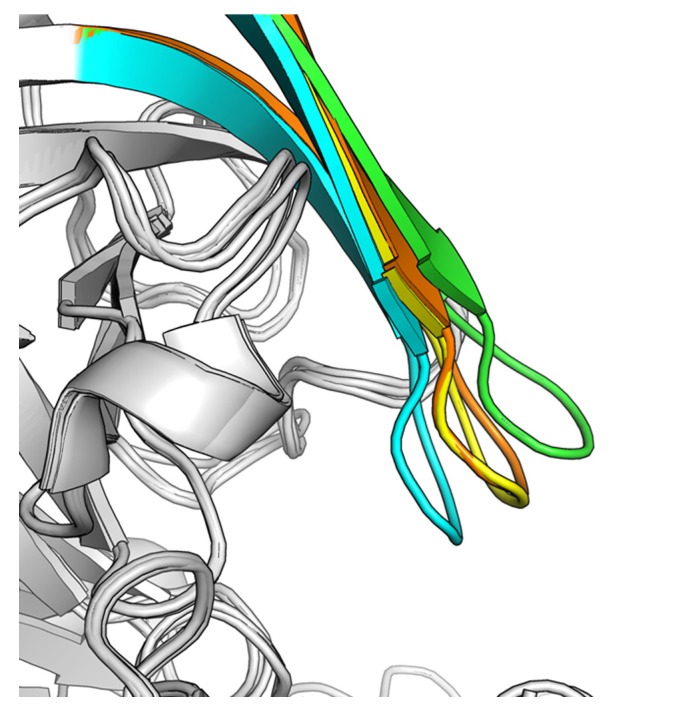
The conformational changes of the flap in BACE1. The superimposition of the X-ray crystal structures of BACE1 represented by pdb code 1FKN (cyan), 1W50 (green), 4IVT (yellow), and 4IVS (brown). The pdb code 4IVT and 4IVS represent the crystal structures of BACE1 complexed with the compounds **19** and **25**, respectively.

As shown in Figure 3A, the acylguanidine of compound **19** formed crucial interactions with two catalytic aspartic acids (Asp228 and Asp32) through three hydrogen bonds. The carboxyl oxygen atoms of the acylguanidine formed water-bridged hydrogen bonds with the side chains of Gln73 and Thr72, and a direct hydrogen bond with Gln73 as well. The substituted benzyl group occupied the S1 subsite of the substrate binding pocket of BACE1. The carboxyl oxygen atom of acetamide also forms a water-bridged hydrogen bond with the nitrogen atom of the amide group of Gln73, while the acetamide nitrogen atom forms another hydrogen bond directly with the main chain carboxyl oxygen of Phe108. The two chlorine atoms may force the acetamide group adopting a perpendicular angle with respect to the benzyl group, which plays an important role in binding interactions between acetamide and BACE1. Besides, the hydrogen bonds of the inhibitor with residues Gln73 and Thr72 induce a semi-closed conformation according to its close and open conformation shown in the structure of BACE1 represented by pdb code 1FKN and 1W50, respectively (Figure 4) [31]. Such a conformation of the flap further strengthens the ligand binding to the enzyme. The indole group pointed toward the back of the S1’ pocket forming a cation-π interaction with Arg235, which appears to contribute further interactions to improve the potency of the inhibitor. 

Following the establishment of the increased potency of acylguanidines with benzyl group substituents, a second round of optimization was conducted to prepare additional indole analogs. The general synthesis route of these compounds was same as that of the previously described compounds (Scheme 2).

As shown in Table 2, all compounds with various substituted indoles exhibited better potency than the initial indole compound **19** (IC_50_ = 1.01 µM). The 6-substituted indole analogs (compounds **23**, **25**, **27**) were slightly more potent than 5-substituted indole analogs (compounds **22**, **24**, **26**). Nitrile group was the optimal substitutent on indole, especially, when the nitrile group was introduce into the 6-position of indole (compound **25**), the most potent analog was identified, which was nearly 23-fold more potent than compound **19** and >1,000-fold more potent than the fragment hit resulting from virtual screening (compound **4**, IC_50_ > 100 µM). 

To further rationalize the ligand binding to BACE1, **25**, the most potent compound in this series, was selected to soak it into the apo crystal of BACE1 and the complex structure was solved at a resolution of 2.47 Å (Figure 3B,D). The superimposition of structures of BACE1 in complex with compounds **19**, **25** and the initial docked fragment are also shown (Figure 3E). As shown in Figure 3B, ligand **25** is situated in a similar position as ligand **19** in the binding pocket of BACE1. The guanidine group again forms critical H-bonding interactions with the two catalytic residues Asp32 and Asp228. The substituted nitrile group forms a new hydrogen bond with Ser328, which may account for the improvement of its binding affinity. This subpocket located at the opposite of flap loop, which is under-explored in previous studies on the discovery of BACE1 inhibitors. The SAR study in the present work further demonstrates its value for future inhibitor design. 

**Table 2:** BACE-1 inhibitory activities of compounds **22**–**28**. 
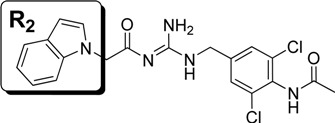

Comp.	R_2_	Inhibition % (100 µM)	IC_50_ (µM)
19	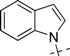	100	1.010 ± 0.091
22	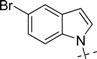	108.7	0.106 ± 0.013
23	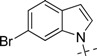	100	0.079 ± 0.008
24	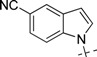	99.5	0.057 ± 0.004
25	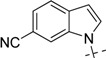	100	0.044 ± 0.005
26	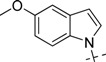	100	0.450 ± 0.058
27	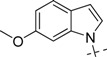	103.2	0.224 ± 0.033
28	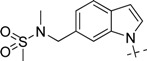	100	0.840 ± 0.101

## 3. Experimental

### 3.1. Chemistry

#### 3.1.1. General Methods

The ^1^H-NMR spectra (300 MHz or 400 MHz) were recorded on Varian Mercury-300 or 400 High Performance Digital FT-NMR instruments using tetramethylsilane as an internal standard. Abbreviations for peak patterns in NMR spectra: br = broad, s = singlet, d = doublet, and m = multiplet. Low-resolution mass spectra were obtained with a Finnigan LCQ Deca XP mass spectrometer using a CAPCELL PAK C18 (50 mm × 2.0 mm, 5 µM) or an Agilent ZORBAX Eclipse XDB C18 (50 mm × 2.1 m, 5 µM) in positive or negative electrospray mode. Purity of all compounds was determined by analytical Gilson high-performance liquid chromatography (HPLC) using an YMC ODS3 column (50 mm × 4.6 mm, 5 µM). Conditions were as follows: CH_3_CN/H_2_O eluent at 2.5 mL min^−1^ flow [containing 0.1% trifluoroacetic acid (TFA)] at 35 °C, 8 min, gradient 5% CH_3_CN to 95% CH_3_CN, monitored by UV absorption at 214 nm and 254 nm. All reagents are of analytical grade pure and used without further purification.

#### 3.1.2. Preparation of *N*-Carbamimidoyl-2-(1*H*-indol-1-yl)acetamide (**5**)

To a solution of indole **6** (1.17 g, 10 mmol) in acetone (30 mL) was added potassium carbonate (2.76 g, 20 mmol) and ethyl bromoacetate **7** (1.65 mL, 15 mmol). The mixture was heated to reflux overnight and then the solvent was concentrated under reduced pressure. Water (100 mL) was added to the residue and the solution was extracted with EtOAc twice (100 mL × 2) and the combined organic layers were washed by turns with dilute HCl, saturated NaHCO_3_ and saturated brine (100 mL × 2). Then the organic layers were dried over Na_2_SO_4_, filtered and evaporated to give a residue, which was purified by silica gel chromatography to afford compound **8** as a yellow amorphous solid (1.53 g, 75.3%). ^1^H-NMR (300 MHz, CD_3_OD): δ 7.67–7.64 (d, *J* = 8.1 Hz, 1H), 7.27–7.24 (m, 2H), 7.20–7.11 (m, 2H), 6.59–6.58(d, *J* = 7.2 Hz, 1H), 4.85 (s, 2H), 4.24–4.21 (q, *J* = 5.1 Hz, 2H), 1.29–1.25 (t, *J* = 5.1 Hz, 3H); ESI: *m/z* 204.1 [M+H]^+^.

To a solution of compound **8** (1.50 g, 7.4 mmol) in THF/EtOH/H_2_O = 2/2/1 mixed solvent (50 mL) was added NaOH (600 mg, 15 mmol). The mixture was stirred at room temperature overnight. Then the mixture was acidified with diluted HCl and extracted with EtOAc. The combined organic layer was concentrated to afford 2-(1*H*-indol-1-yl)acetic acid. To a solution of the crude acid product (175 mg, 1 mmol) in DMF (10 ml) was added HATU (570 mg, 1.5 mmol), NMM (224 μL, 2 mmol), and Boc-guanidine (190 mg, 1.2 mmol). The mixture was stirred at room temperature overnight. Water (50 mL) was added into the reaction mixture and the solution was extracted with EtOAc (50 mL × 2) and the combined organic layers were washed by turns with dilute HCl, saturated NaHCO_3_, and saturated brine twice, then the organic layers were dried over Na_2_SO_4_, filtered and evaporated to give a residue which was purified by silica gel chromatography to afford compound **9** as a yellow amorphous solid (250 mg, 79.1%). Compound **9** (250 mg, 0.79 mmol) was deprotected by 4M HCl in dioxane (20 mL), then concentrated to afford compound **5**. ^1^H-NMR (300 MHz, CD_3_OD): δ 7.65–7.62 (d, *J* = 7.5 Hz, 1H), 7.27–7.19 (m, 2H), 7.14–7.08 (m, 2H), 6.56 (s, 1H), 4.79 (s, 2H); ESI: *m/z* 216.9 [M+H]^+^.

#### 3.1.3. General Procedure for the Preparation of Indole Acylguanidine Analogs **12**–**28**

To a solution of 2-(1*H*-indol-1-yl)acetic acid (175 mg, 1 mmol) in DMF (10 mL) was added HATU (570 mg, 1.5 mmol), NMM (224 μL, 2 mmol) and (*Z*)-*tert*-butylamino(methylthio)methylene carbamate (228 mg, 1.2 mmol). The mixture was stirred at room temperature overnight. Water (50 mL) was added to the reaction mixture, the solution was extracted with EtOAc (50 mL × 2) and the combined organic layers were washed by turns with dilute HCl, saturated NaHCO_3_, and saturated brine twice. Then the organic layers were dried over Na_2_SO_4_, filtered and evaporated to give a residue, which was purified by silica gel chromatography to afford compound **10** as a yellow amorphous solid (310 mg, 89.3%). ^1^H-NMR (300 MHz, CDCl_3_): δ 7.66–7.64 (d, *J* = 7.8 Hz, 1H), 7.26–7.10 (m, 4H), 6.57 (s, 1H), 4.97 (s, 2H), 2.16 (s, 3H), 1.46 (s, 9H); ESI: *m/z* 347.9 [M+H]^+^.

To a solution of compound **10** (86 mg, 0.25 mmol) in DCM (25 mL) was added benzylamine (55 μL, 0.5 mmol) and triethylamine (90 μL, 0.5 mmol). The mixture was stirred at room temperature overnight. The solvent was evaporated to give a residue which was purified by silica gel chromatography to afford compound **11**. Compound **11** was deprotected by 4 M HCl in dioxane (20 mL), then concentrated to afford compound **12** as a colorless amorphous solid (61 mg, 83.7%) ^1^H-NMR (300 MHz, CDCl_3_): δ 7.66–7.63 (d, *J* = 8.1Hz, 1H), 7.33–7.26 (m, 4H), 7.23–7.08 (m, 5H), 6.56 (s, 1H), 4.87 (s, 2H), 4.50–4.48 (d, *J* = 6.0 Hz, 2H); EI: *m/z* 306[M]^+^. The following compounds were similarly prepared:

*2-(1H-Indol-1-yl)-N-(N-(3-iodobenzyl)carbamimidoyl)acetamide* (**13**): Yellow amorphous solid, yield: 53.3%. ^1^H-NMR (300 MHz, CDCl_3_): δ 7.64–7.59 (m, 3H), 7.31–7.17 (m, 2H), 7.17–7.02 (m, 4H), 6.55 (s, 1H), 4.85 (s, 2H), 4.38–4.36 (d, *J* = 6.6Hz, 2H); EI: *m/z* 432[M]^+^.

*2-(1H-Indol-1-yl)-N-(N-(3-methoxybenzyl)carbamimidoyl)acetamide* (**14**): Yellow amorphous solid, yield: 61.4%. ^1^H-NMR (300 MHz, CDCl_3_): δ 7.64–7.61 (d, *J* = 7.8 Hz, 1H), 7.31–7.19(m, 3H), 7.16–7.03 (m, 2H), 6.85–6.77 (m, 3H), 6.55 (s, 1H), 4.86 (s, 2H), 4.48–4.47 (d, 2H), 3.82 (s, 3H); ESI: *m/z* 337.0 [M+H]^+^.

*N-(N-(3,5-Difluorobenzyl)carbamimidoyl)-2-(1H-indol-1-yl)acetamide* (**15**): Yellow amorphous solid, yield: 46.4%. ^1^H-NMR (300 MHz, CDCl_3_): δ 7.63–7.60 (d, *J* = 7.8 Hz, 1H), 7.28–7.25(d, *J* = 7.6 Hz, 1H), 7.20–7.15 (t, *J* = 8.1 Hz, 1H), 7.11–7.05 (m, 2H), 6.73–6.66 (m, 3H), 6.53 (s, 1H), 4.83 (s, 2H), 4.39–4.37 (d, *J* = 6.0 Hz, 2H); ESI: *m/z* 342.9 [M+H]^+^.

*2-(1H-Indol-1-yl)-N-(N-(3-(trifluoromethoxy)benzyl)carbamimidoyl)acetamide* (**16**): Colorless amorphous solid, yield: 54.1%. ^1^H-NMR (300 MHz, CDCl_3_): δ 7.63–7.60 (d, *J* = 8.4 Hz, 1H), 7.34–7.20 (m, 2H), 7.18–7.02 (m, 6H), 6.53 (s, 1H), 4.82 (s, 2H), 4.46–4.44 (d, *J* = 6.6 Hz, 2H); ESI: *m/z* 390.0 [M+H]^+^.

*N-(N-(3,5-Dichlorobenzyl)carbamimidoyl)-2-(1H-indol-1-yl)acetamide* (**17**): Yellow amorphous solid, yield: 66.1%. ^1^H-NMR (300 MHz, CDCl_3_): δ 7.62–7.60 (d, *J* = 8.1 Hz, 1H), 7.28–7.25 (m, 2H), 7.20–7.15 (t, 1H), 7.10–7.04 (m, 4H), 6.53 (s, 1H), 4.83 (s, 2H), 4.34–4.32 (d, *J* = 6.3 Hz, 2H); EI: *m/z* 374.

*N-(N-(4-Acetamidobenzyl)carbamimidoyl)-2-(1H-indol-1-yl)acetamide* (**18**): Colorless amorphous solid, yield: 46.1%. ^1^H-NMR (300 MHz, CDCl_3_): δ 7.64–7.61 (d, *J* = 7.8 Hz, 1H), 7.43–7.40 (d, *J* = 8.1 Hz, 2H), 7.31–7.19 (m, 3H), 7.12–7.07 (m, 3H), 6.54 (s, 1H), 4.85(s, 2H), 4.42–4.40 (d, *J* = 6.0 Hz, 2H), 2.18(s, 3H); ESI: *m/z* 364.0 [M+H]^+^.

*N-(N-(4-Acetamido-3,5-dichlorobenzyl)carbamimidoyl)-2-(1H-indol-1-yl)acetamide* (**19**): Yellow amorphous solid, yield: 36.8%. ^1^H-NMR (300 MHz, CDCl_3_): δ 7.62–7.59 (d, *J* = 7.8 Hz, 1H), 7.27–7.25 (m, 2H), 7.19–7.16 (m, 3H), 7.09–7.07 (m, 2H), 6.53 (s, 1H), 4.83 (s, 3H), 4.33–4.30 (d, *J* = 6.2 Hz, 2H), 2.26 (s, 3H); ESI: *m/z* 431.9 [M+H]^+^.

*N-(N-(3,5-Dichloro-4-(ethylamino)benzyl)carbamimidoyl)-2-(1H-indol-1-yl)acetamide* (**20**): Colorless amorphous solid, yield: 24.8%. ^1^H-NMR (300 MHz, CDCl_3_): δ 7.63–7.60(d, *J* = 8.4 Hz, 1H), 7.30–7.25 (m, 2H), 7.18–7.16 (t, *J* = 7.8 Hz, 1H), 7.11–7.06 (m, 2H), 6.55 (s, 1H), 4.85 (s, 2H), 4.29–4.27 (d, *J* = 6.8 Hz, 2H), 3.43–3.36 (q, *J* = 7.5 Hz, 2H), 1.25–1.20 (t, *J* = 7.5 Hz, 3H); ESI: *m/z* 417.9 [M+H]^+^.

*N-(4-((3-(2-(1H-Indol-1-yl)acetyl)guanidino)methyl)-2,6-dichlorophenyl)isobutyramide* (**21**): Color-less amorphous solid, yield: 49.6%. ^1^H-NMR (300 MHz, CDCl_3_): δ 7.62–7.59 (d, *J* = 7.8 Hz, 1H), 7.27–7.25 (m, 3H), 7.20–7.16 (m, 2H), 7.09–7.05 (m, 2H), 6.53–6.52 (d, *J* = 6.6 Hz, 1H), 4.83(s, 2H), 4.33–4.31 (d, 2H), 2.67–2.61 (m, 1H), 1.34–1.32 (d, *J* = 6.9 Hz, 6H); ESI: *m/z* 460.0 [M+H]^+^.

*N-(N-(4-Acetamido-3,5-dichlorobenzyl)carbamimidoyl)-2-(5-bromo-1H-indol-1-yl)acetamide* (**22**): Colorless amorphous solid, yield: 37.5%. ^1^H-NMR (300 MHz, CDCl_3_): δ 7.68(s, 1H), 7.23–7.20(d, *J* = 7.8 Hz, 1H), 7.11–7.07 (m, 4H), 6.43 (s, 1H), 4.77 (s, 2H), 4.30–4.28 (d, *J* = 6.9 Hz, 2H), 2.26 (s, 3H); ESI: *m/z* 509.9 [M+H]^+^.

*N-(N-(4-Acetamido-3,5-dichlorobenzyl)carbamimidoyl)-2-(6-bromo-1H-indol-1-yl)acetamide* (**23**): Colorless amorphous solid, yield: 57.8%. ^1^H-NMR (300 MHz, CDCl_3_): δ 7.46–7.41 (m, 2H), 7.26–7.14 (m, 3H), 7.05 (s, 1H), 6.49 (s, 1H), 4.77 (s, 2H), 4.34–4.32(d, *J* = 6.9 Hz, 2H), 2.25 (s, 3H); ESI: *m/z* 509.9 [M+H]^+^.

*N-(N-(4-Acetamido-3,5-dichlorobenzyl)carbamimidoyl)-2-(5-cyano-1H-indol-1-yl)acetamide* (**24**): Colorless amorphous solid, yield: 38.8%. ^1^H-NMR (300 MHz, CDCl_3_): δ 7.87(d, *J* = 8.1 Hz, 1H), 7.38–7.35 (d, *J* = 7.8 Hz. 1H), 7.27–7.24 (m, 3H), 7.18 (s, 1H), 6.98 (s, 2H), 6.58 (s, 1H), 4.82 (s, 2H), 4.25–4.23 (d, *J* = 6.0 Hz, 2H), 2.27 (s, 3H); ESI: *m/z* 456.9 [M+H]^+^.

*N-(N-(4-Acetamido-3,5-dichlorobenzyl)carbamimidoyl)-2-(6-cyano-1H-indol-1-yl)acetamide* (**25**): Colorless amorphous solid, yield: 53.9%. ^1^H-NMR (300 MHz, CDCl_3_): δ 7.66–7.63 (d, *J* = 7.8 Hz, 1H), 7.48 (s, 1H), 7.30–7.26 (m, 1H), 7.13 (s, 3H), 6.59–6.58 (d, *J* = 6.9 Hz, 1H), 4.83 (s, 2H), 4.30–4.28 (d, *J* = 6.0 Hz, 2H), 2.26 (s, 3H); ESI: *m/z* 456.9 [M+H]^+^.

*N-(N-(4-Acetamido-3,5-dichlorobenzyl)carbamimidoyl)-2-(6-methoxy-1H-indol-1-yl)acetamide* (**26**): Colorless amorphous solid, yield: 32.1%. ^1^H-NMR (300 MHz, CDCl_3_): δ 7.47–7.45 (d, *J* = 8.4 Hz, 1H), 7.27 (s, 2H), 6.96 (s, 1H), 6.76–6.70 (m, 3H), 6.45–6.44 (d, *J* = 7.2 Hz, 1H), 4.75 (s, 3H), 4.34–4.32 (d, *J* = 6.3 Hz, 2H), 3.83 (s, 3H), 2.26 (s, 3H); ESI: *m/z* 461.9 [M+H]^+^.

*N-(N-(4-Acetamido-3,5-dichlorobenzyl)carbamimidoyl)-2-(6-methoxy-1H-indol-1-yl)acetamide* (**27**): Colorless amorphous solid, yield: 40.7%. ^1^H-NMR (300 MHz, CDCl_3_): δ 7.47–7.45 (d, *J* = 8.1 Hz, 1H), 7.27 (s, 2H), 6.96 (s, 1H), 6.76–6.70 (m, 3H), 6.45–6.44 (d, *J* = 7.2 Hz, 1H), 4.75 (s, 3H), 4.34–4.32 (d, *J* = 6.4 Hz, 2H), 3.83 (s, 3H), 2.26 (s, 3H); ESI: *m/z* 461.9 [M+H]^+^.

*N-(N-(4-Acetamido-3,5-dichlorobenzyl)carbamimidoyl)-2-(6-((N-ethylmethylsulfonamido)methyl)-1H-indol-1-yl)acetamide* (**28**): Yellow amorphous solid, yield: 48.4%. ^1^H-NMR (300 MHz, CDCl_3_): δ 7.59–7.56 (d, *J* = 8.1 Hz,1H), 7.14 (s, 3H), 7.10 (s, 1H), 7.03–7.00 (d, *J* = 7.8 Hz, 1H), 6.53–6.52(d, *J* = 7.2 Hz, 1H), 4.82 (s, 2H), 4.32 (s, 4H), 2.80 (s, 3H), 2.72 (s, 3H), 2.25 (s, 3H); ESI: *m/z* 552.9 [M+H]^+^.

### 3.2. Virtual Screening

Fragment subset of ZINC database was downloaded from its web site [37]. This fragment library was processed by the molconvert program in the ChemAxon software package to convert the Smiles format into 3D SDF files [38]. Then the Ligprep workflow in the Schrödinger software was used to minimize and generate ligand structures suitable for the docking study [38]. Two crystal structures (1FKN and 3IGB) were prepared by following the default parameters in the Protein Preparation Wizard [39]. Then the Glide program was used to generate the grid files, and the Glide VS module was executed to screen the fragment library described earlier. The 2000 fragments obtained from Glide VS docking were further docked into two binding sites with the Glide SP module. Finally, the 100 top-scoring fragments were selected for manual check and five fragments with feasible binding modes were purchased for enzymatic assay (purchased from ChemDiv, San Diego, CA, USA; ID: 6179-0008, 1545-0107, G373-3345, G793-0250, 8016-8644). 

### 3.3. Fluorescence Resonance Energy Transfer (FRET) Based Enzymatic Assay

The BACE1 fluorescence resonance energy transfer assay kit was purchased from Invitrogen (Carlsbad, CA, USA, cat. P2985). BACE1 activity assays were carried out following the manufacturer’s protocol. The enzyme, substrate, and compounds were diluted in a reaction buffer (50 mM sodium acetate, pH 4.5) to make 3× working solutions. The assay was carried out in a black 384-well microplate with a final volume of 30 μL which contains each 10 μL of 3× substrate, enzyme, and compound stocks, respectively. The final concentration of DMSO is less than 3% (v/v). The reaction mixture was incubated at 37 °C for 90 min with oscillation. Then, 10 μL of a stop solution (2.5 M sodium acetate) was added to stop the reaction. Finally, the fluorescent intensity of the enzymatic product was measured at Ex/Em = 535 nm/585 nm on a BioTek SYNERGY4 reader (BioTek, Winooski, VT, USA). Triplicate was run for each assay. IC_50_ values were calculated from the concentration–inhibition curves by nonlinear regression analysis. Eight concentrations were tested for each compound to produce the concentration–inhibition curve.

### 3.4. BACE1 Protein Expression and Crystallization

#### 3.4.1. Protein Purification and Crystallization

A detailed description of the production of recombinant human BACE1 has been provided in our previous publication [40]. Briefly, BACE1 was expressed in *E. coli* as inclusion bodies that were then denatured and refolded into the active monomer. A cDNA fragment encoding BACE1 residues 43-454 was cloned into pET28a with a TEV protease cleavage site following a six-residue His-Tag added at the N-terminus. After refolding, nickel beads (Ni Sepharose^TM^ High Performance, Amersham Biosciences, Uppsala, Sweden) were used to concentrate the protein. The eluted BACE1 was then injected into a 124 mL Hiload Superdex75 column from which it was eluted with 1 mM DTT/0.5 M urea/150 mM NaCl/20 mM Tris-HCl, pH 7.5. In order to obtain crystals with different protein packing patterns, two mutations, K75A and E77A, were introduced into BACE1 together. The active BACE1 monomer in the elution buffer was concentrated to 8–10 mg/mL. Crystallization of apo-mutated BACE1 was performed by mixing equal volumes of the protein stock solution and the precipitant (1.7 M Li2SO4/100 mM HEPES, pH 7.5). To obtain a crystalline complex with compounds 19 and 25, a 100 mM solution of the compound in DMSO was diluted 20-fold, to 5 mM, in the precipitant solution so as to generate a soaking drop. The crystal was transferred into the soaking drop and left for 24 h prior to data collection. All the crystallization utilized the vapor diffusion method in hanging drops. The perfluoropolyether, PFO-X175/08 (Hampton Research, Aliso Viejo, CA, USA), was used as cryoprotectant for all the crystals.

#### 3.4.2. Structure Determination and Refinement

Data were collected at 100 K on beamline BL17U (at wavelength 0.9791 Å) at the Shanghai Synchrotron Radiation Facility (SSRF, Shanghai, China). The data were processed with the XDS [41] software packages, and the structures were then solved by molecular replacement, using the CCP4 program Phaser [42]. The search model used for the crystals was the mutated type BACE1 structure (PDB code 2B8L). The structures were refined using the program PHENIX [43]. With the aid of the program Coot [44], ligand, water molecules, and others were fitted into the initial *F*_o_-*F*_c_ maps. 

## 4. Conclusions

In summary, with the aid of virtual screening and crystal structures, a series of small molecule inhibitors of BACE1 bearing an indole acylguanidine core have been discovered. Comparing the initial hit more than a 1,000-fold increase in potency of the final lead compound has been achieved. Future study focused on the improvement of potency and optimization of the drug-like properties of this series will pave the way for developing new BACE1 inhibitors against AD.
